# Unsupervised Domain Adaptive 1D-CNN for Fault Diagnosis of Bearing

**DOI:** 10.3390/s22114156

**Published:** 2022-05-30

**Authors:** Xiaorui Shao, Chang-Soo Kim

**Affiliations:** 1 Industrial Science Technology Research Center, Pukyong National University, Busan 608737, Korea; shaoxiaorui@pukyong.ac.kr; 2 Information Systems, Pukyong National University, Busan 608737, Korea

**Keywords:** fault diagnosis, vibration signal, 1D-CNN, domain adaption, autoencoder

## Abstract

Fault diagnosis (FD) plays a vital role in building a smart factory regarding system reliability improvement and cost reduction. Recent deep learning-based methods have been applied for FD and have obtained excellent performance. However, most of them require sufficient historical labeled data to train the model which is difficult and sometimes not available. Moreover, the big size model increases the difficulties for real-time FD. Therefore, this article proposed a domain adaptive and lightweight framework for FD based on a one-dimension convolutional neural network (1D-CNN). Particularly, 1D-CNN is designed with a structure of autoencoder to extract the rich, robust hidden features with less noise from source and target data. The extracted features are processed by correlation alignment (CORAL) to minimize domain shifts. Thus, the proposed method could learn robust and domain-invariance features from raw signals without any historical labeled target domain data for FD. We designed, trained, and tested the proposed method on CRWU bearing data sets. The sufficient comparative analysis confirmed its effectiveness for FD.

## 1. Introduction

Bearing is one of the critical elements for the rotating machine in the production industries, the healthy status of which significantly influences production efficiency [1,2], system safety, and reliability [3]. Therefore, designing an advanced approach for fault diagnosis (FD) of bearing is critical, aiming to identify and detect the failure of bearing before it happens.

The current methods for FD mainly include model-based, signal-based, and learning-based methods [4,5]. Among them, model-based and signal-based methods require a priori knowledge of the operating systems, which increases the difficulty and cost for FD. On the contrary, learning-based methods do not require prior system knowledge and have extracted more attention.

Learning-based methods for FD can be divided into traditional machine learning and deep learning methods. Traditional learning methods consist of support vector machine (SVM) [6], decision tree (DT) [7], and random forest (RF) [8]. Primarily, SVM aims at finding one segmentation hyperplane with various kernels, which can correctly classify the data and maximize the spacing among various faults. For example, Diego, et al. [9] proposed an automatic scheme based on SVM for FD of bearing, where only normal vibration signals are used. Lu and Li [10] utilized principal component analysis (PCA) and SVM for FD of bearing. DT is a tree-based method that finds the critical feature to construct the rule for FD. For example, Tan, et al. [11] utilized DT to detect the fault of an induction motor, and Asman, et al. [12] applied it to find the causes in a 275 kV transmission line network. RF integrates multiple DTs to select the key features and make the decision for FD [13,14]. It usually performs better than DT. However, there are some limitations that occur in the traditional machine learning methods. For instance, the performance of SVM highly depends on kernel selection [15]. DT and RF are sensitive to noisy data and still require selecting valuable features by hand [16].

Compared to the traditional machine learning methods, deep learning-based methods extract rich hidden features from raw vibration signals without any handcraft feature selection operations and have been widely adopted. Specifically, convolution neural network (CNN) [17], one of the deep neural networks, is the mainstream choice for FD due to its excellent feature extraction capacity. For example, Chen, et al. [18] applied two-dimensional (2-D) CNN for FD of the gearbox, where vibration signals are processed into statistical measures and frequency domain representations by fast Fourier transform (FFT) as CNN’s input. Wen, et al. [19] also adopted 2-D CNN based on LeNet to extract the hidden patterns from 2-D images for FD. Shao, et al. [20] applied a pre-trained 2-D CNN structure VGG-16 [21] for FD, in which raw signals are processed into the 2-D image using continuous wavelet transformation (CWT). However, 2-D CNN requires more time to train the model and give the results of FD due to the usage of extra preprocessing operations. Its performance is affected by different processing methods. In contrast, 1D-CNN can overcome those drawbacks and has been applied for FD with raw signals. For example, Zhang proposed [22] a novel 1D-CNN with wide convolution operation (WDCNN) at the first layer for FD of bearing. The experimental analysis confirmed its good anti-noise and domain adaption capacities when combining adaptive batch normalization (AdaBN) [23]. Jiang, et al. [24] proposed a multi-scale CNN (MSCNN) for FD of the wind turbine gearbox, in which the raw signal is split into multi-course grained features with the average operation, and then multi-scale 1D-CNN is used to learn differ-scale hidden features. The experimental results suggested that three-scale CNN is better for FD, considering the diagnostic performance and required time. Motivated by MSCNN, Shao, et al. [25] proposed a multi-scale feature fusion CNN (MSFFCNN) for 1-D time series classification which can be used for FD. Moreover, Kim, et al. [5] applied multi-domain features, including statistical, raw signals and discrete wavelet transformed (DWT) frequency domain features as the input of 1D-CNN (MDCNN) for FD of bearing. Liu, et al. [26] combined 1D-CNN and multi-task learning for FD of wheelset bearing, in which the signals’ loadings and speed identification were measured to make full use of information to improve FD performance.

Although the above 1D-CNN-based methods have achieved good performance within the tolerable processing time, they still have limitations. Firstly, they require sufficient labeled historical data to train the model. However, it is difficult or even not available to collect all kinds of faulty data in practice due to the signals’ randomness and irregularity. Secondly, they assume that both training (source domain) and testing (target domain) data have the same distribution while they are quite different, and as a result, the performance is unsatisfactory and still can be improved.

Luckily, deep transfer learning (DTL) can learn and transfer the knowledge from the source domain into the target domain with limited training samples that have been applied to FD. The DTL-based method for FD is relatively new. It mainly consists of supervised pre-trained DTL and unsupervised DTL (also called domain adaption). Supervised pre-trained DTL trains the model on the source domain data while fine-tuning the model on the target domain data. For example, Shao, et al. [20] trained the VGG-16 on the ImageNet set and fine-tuned the model for each faulty data set for FD. Brusa, et al. [27] pre-trained the YAMNet on the sound and music data set and fine-tuned the model on the bearing data set for FD. Yao, et al. [28] pre-trained the deep model on the source domain faulty data and froze some parts to fine-tune one CNN on the target domain data for FD of nuclear power plants. Unlike supervised pre-trained DTL, unsupervised DTL for FD does not require any labeled target domain data. It aims to reduce the domain shift between two domains by calculating their similarities. For example, Lu, et al. [29] utilized a fully connected neural network to extract the hidden features from two domains. Moreover, the single-kernel maximum mean discrepancy (MMD) was used to minimize the domain shift for FD. Wen, et al. [30] combined a three-layer sparse autoencoder and single-kernel MMD for FD of bearing. Zhu, et al. [31] utilized 2-D CNN to extract hidden features and multi-kernel MMD at the last two layers to reduce the domain shifts for FD. However, MMD has serval shortcomings, such as sensitivity to kernel selection and data samples, and low scalability for large applications [32]. To overcome MMD’s shortcomings, Wang, et al. [32] utilized correlation alignment (CORAL) [33,34] to calculate the distribution discrepancy between each layer of stacked denoising autoencoder for FD of a power plant thermal system. However, calculating each layer’s CORAL is time-consuming, and we argue that CNN can extract more robust features than a fully connected neural network.

As we described above, few references have utilized 1D-CNN to extract hidden features from raw signals considering the domain shift for FD. This manuscript proposed a novel lightweight framework for FD of bearing. A 1D-CNN autoencoder was designed to extract rich features with less noise from raw vibration signals. Furthermore, the domain shifts were minimized by calculating the CORAL distance between extracted source and target features. Sufficient experiments confirmed that the proposed method could detect faults in a complex environment accurately.

The main contribution of this manuscript is summarized as follows:

- One new lightweight domain adaption 1D-CNN autoencoder was proposed. It could automatically learn rich, robust hidden features from raw vibration signals for FD with less time.
- The CORAL was combined with the proposed 1D-CNN autoencoder to minimize the domain shift between source and target domains. Therefore, the proposed method could learn rich, robust, and domain-invariant hidden features to accurately and timely detect the cross-domain faults without labeled target domain samples. Sufficient comparative experiments confirmed its effectiveness.
- Each component’s effectiveness was analyzed through one ablation study.
- The effectiveness of the reconstruction ratio is discussed in depth.

The rest of this manuscript is arranged as follows. Section 2 gives some pre-knowledge used in the proposed method, including the problem statement, CNN, and CORAL distance. Section 3 describes the proposed method for FD in detail. The experimental verification is carried out in Section 4. Section 5 discusses the proposed method for FD. At last, we conclude this manuscript in Section 6.

## 2. Preliminaries

### 2.1. Problem Statement

Assume that we collected some data in the source domain, denoted as $D^{𝓈}={\left\{\left(x_{i}^{s},y_{i}^{s}\right)\right\}}_{i=1}^{N}$. Where $x_{i}^{s}=\left\{t_{i,j}^{s},t_{i,j+1}^{s},\ldots ,t_{i,n+j-1}^{s}\right\}\in X^{s}\in R^{n}$ is the vibration signal, which consists of n data points from time step j to n+j−1. Each signal $x_{i}^{s}$ corresponds to one faulty type $y_{i}^{s}=\left\{1,2,3,\ldots ,C\right\}\in Y^{s}$. Same for the source domain data, we collected target domain data $D^{t}={\left\{\left(x_{i}^{t}\right)\right\}}_{i=1}^{N}$ without labels, where $x_{i}^{t}=\left\{t_{i,j}^{t},t_{i,j+1}^{t},\ldots ,t_{i,n+j-1}^{t}\right\}\in X^{t}\in R^{n}$. We assumed that both source and target domain data have the same feature space X and label space Y but different distributions, i.e., $X^{s}=X^{t}=X$, $Y^{s}=Y^{t}=Y$ and $P(X^{s})\neq P\left(X^{t}\right)$. Our goal was to build one transferable model $f\left(\cdot \right)$, which can learn domain-invariant features by using labeled source domain samples and unlabeled target samples that enable: X→Y. In this manuscript, we first utilized one 1D-CNN autoencoder to learn hidden features with less noise from source and target data; then, the extracted features were processed by CORAL to minimize the distribution discrepancy. Therefore, the proposed method could learn common domain-invariant features to detect the fault on unseen target domain sets accurately.

### 2.2. CNN

CNN is a classical feedback neural network which has been widely used in the area of image classification [17,35], medical heathy management and control [36,37], and time series processing [38,39,40] due to its robust feature extraction capacity. The conventional CNN mainly is used to process 2-D images, while this manuscript is for 1-D signals. Giving one signal x, the 1D-CNN learns the hidden patterns through three key components. Firstly, convolution operation with various kernels is calculated to extract features in multiple views. The convoluted features have parameter sharing and local connection characteristics, which ensures that the neural network extracts rich hidden features with fewer parameters than a fully connected neural network. Then, one activation function activates the convoluted features to strengthen the non-linear expression. Those two processes are shown in (1), where the $k^{th}$ feature map $h_{k}$ is calculated by using the convolution operation * between signal x and $k^{th}$ kernel filter $f_{k}$ with a bias $b_{k}$; σ is one activation function such as the sigmoid and rectified linear unit (ReLU) [41]. Thirdly, the pooling operation reduces the feature map’s size to speed up the network. The most used pooling operation is the maximum polling operation max, which calculates the maximum value of the feature map within a certain range w, as shown in (2). The final features h are obtained after n-time convolution and maximum pooling operations (called convolution block ConvBlock), as shown in (3). The final features h are used for predicting the faults. More details about CNN can be found in [17].

(1) $$h_{k}=\sigma \left(x*f_{k}+b_{k}\right)$$

(2) $$h_{pk}=max\left(h_{k},w\right)$$

(3) $$h=ConvBlock_{n}\left(x\right)=max{\left(\sigma \left(x*f_{k}+b_{k}\right)\right)}_{n}$$

### 2.3. CORAL Distance

CORAL [33] calculates the source and target features’ second-order statistics (covariances) to align their distributions. It is a parameter-free way to measure the distance between source and target domains and does not require any labeled target domain samples. Compared to MMD, it is not sensitive to kernel selection and data samples, which is suitable for vibration signal data. The calculation of CORAL is denoted in (4), where $C_{s}$ and $C_{t}$ are second-order statistics of source and target features, respectively; d is the dimension of source and target features; and ${\left\|\cdot \right\|}_{F}^{2}$ denotes the square matrix of Frobenius norm. The covariance matrix is calculated as (5) and (6), where $n_{s}$ and $n_{t}$ are the numbers of source and target features, respectively; $h_{s}$ and $h_{t}$ denote, respectively, model extracted hidden features for source and target domains; and 1 is a column vector with all elements equal to 1.

(4) $$L_{coral}=\frac{1}{4d^{2}}\|C_{s}-C_{t}{\|}_{F}^{2}$$

(5) $$C_{s}=\frac{1}{n_{s}-1}\left(h_{s}^{T}h_{s}-\frac{1}{n_{s}}{\left(1^{T}h_{s}\right)}^{T}\left(1^{T}h_{s}\right)\right)$$

(6) $$C_{d}=\frac{1}{n_{t}-1}\left(h_{t}^{T}h_{t}-\frac{1}{n_{t}}{\left(1^{T}h_{t}\right)}^{T}\left(1^{T}h_{t}\right)\right)$$

## 3. The Proposed Method

**Figure 1:**
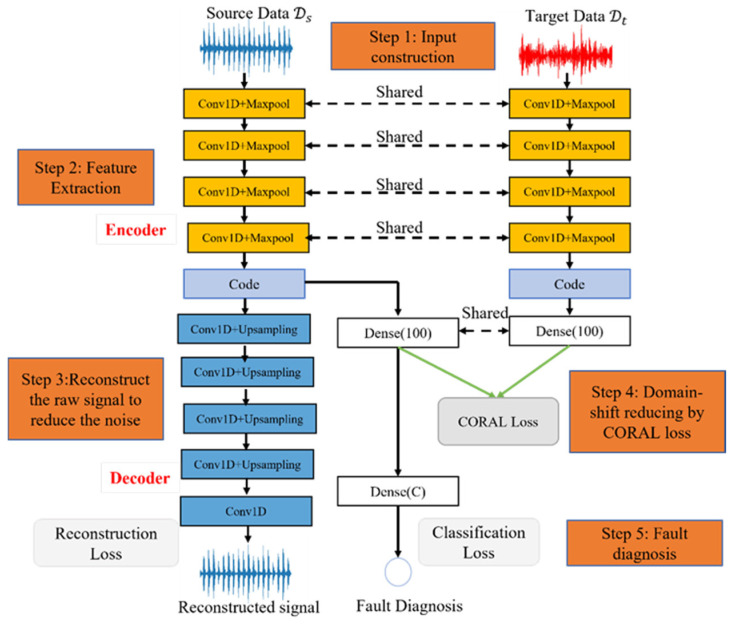
The structure of the proposed method for FD.

The proposed method for FD is shown in Figure 1. It mainly consists of five steps: input construction, feature extraction, reconstruction of the raw signal, domain shift reduction, and fault diagnosis. More details about each part are described in the following subsection.

### 3.1. Input Construction

**Figure 2:**
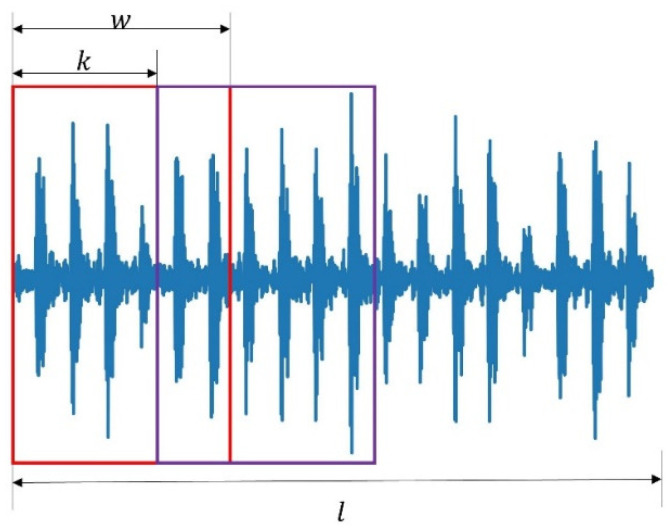
An example of an overlap method to generate training samples.

The proposed method for FD utilizes labeled source domain data and unlabeled target domain data as the input of the 1D-CNN for FD. Moreover, to keep the source domain inner character and reduce the noise, the 1D-CNN autoencoder reconstructs the source domain signal. Thus, the proposed method has dual inputs and outputs, as shown in (7) and (8), where $x_{i}^{s}\;and\;x_{i}^{t}$ are signals from source $D^{𝓈}$ and target $D^{t}$, respectively; N is the number of samples. In practice, we collected one long signal for each kind of faulty data, and the overlap algorithm was applied to construct training samples, as shown in Figure 2. The details of the process for the overlap algorithm are given in Algorithm 1. One long signal Sig with length l could generate $N=⎣\frac{l-w}{k}⎦+1,k\leq w$ samples, where k is the overlap step, w is each sample’s length, and ⎣·⎦ is a round-down operation.

(7) $$Input={\left(x_{i}^{s},x_{i}^{t}\right)}_{i=1}^{N}$$

(8) $$Output={\left(x_{i}^{s},y_{i}^{s}\right)}_{i=1}^{N}$$

**Algorithm 1:** The overlap algorithm to generate training samples.
**Input:** Given one long signal Sig witha length of l, whose fault type is F; the training sample’s number N; and the training sample’s length w. **Output:**
*N* training samples {$x_{i},y_{i}$}. 1. calculating the overlap step $k=⎣\frac{l-w}{N-1}⎦$; defined each sample’s starting index s=0 and ending index e=w; defined Input and Label matrix to save training features $x_{i}$ and corresponding fault type $y_{i}$. 2. **For**
*i* in 1 to l:{ 3.  $Input\left[i\right]=Sig\left[s:e\right]$ 4.  $Label\left[i\right]=F$ 5.  s=s+k 6.  e=s+w} 7. **End for** 8. **Return**
Input and Label

### 3.2. Feature Extraction

The proposed method utilized a 1D-CNN autoencoder to extract hidden features with less noise for source and target data. The encoder was implemented with four stacked convolution blocks (Conv1D + Maxpool) to extract representative features (code), as marked in orange color in Figure 1. The decoder part was used to reduce the noise, as marked in blue color. The term ‘Conv1D’ is the operation of 1-D convolution, and ‘Maxpool’ denotes the maximum pooling operation. We set the small output nodes {16,24,24,24} for four 1-D convolution layers. Moreover, the wide kernel size was adopted for convolution operation since it could mine the short-term and long-term relationships in one long vibration signal [25]. Primarily, we set kernel size {12,9,9,9} for four 1D-CNN layers. Moreover, all layers employed ReLU to activate the hidden feature map to strengthen its non-linear expression. The feature extraction process for source and target data are written as (9) and (10), where raw signals $x_{i}^{s}$ and $x_{i}^{t}$ are fed into four convolution blocks $ConvBlok_{4}$ to extract the hidden features $Code_{s}$ and $Code_{t}$.

(9) $$Code_{s}=ConvBlok_{4}\left(x_{i}^{s}\right)$$

(10) $$Code_{t}=ConvBlok_{4}\left(x_{i}^{t}\right)$$

### 3.3. Reconstruct the Raw Signal

The above step extracted hidden features. However, we believed it still contained some noise, which may significantly influence the performance of FD. Therefore, the proposed method adopted one decoder to reconstruct the signal to reduce the noise for source domain data. We did not adopt a decoder for target data (1) because only source features are used for FD and the noise in the target features do not influence its effectiveness but adopting a decoder in the target data increases the model’s size, and (2) to keep source features’ self-domain characters uninfluenced by target features.

We adopted one symmetrical structure with an encoder to reconstruct the source signal $x_{i}^{s^{\prime }}$, as marked with blue color in Figure 1, where the term ‘Upsampling’ is the upsampling operation, and this step could be written as (11). To obtain the near-optimal reconstructed signal $x_{i}^{s^{\prime }}$ with less noise, the model calculates the mean square error (MSE) loss between raw signal $x_{i}^{s}$ and reconstructed signal $x_{i}^{s^{\prime }}$ to update the network with a gradient descent algorithm, denoted as (12).

(11) $$x_{i}^{s^{\prime }}=Decoder\left(Code_{s}\right)$$

(12) $$L_{mse}\left(x_{i}^{s},x_{i}^{s^{\prime }}\right)=\frac{1}{N}\overset{N}{\underset{i=1}{\sum }}{\left(x_{i}^{s}-x_{i}^{s^{\prime }}\right)}^{2}$$

### 3.4. Domain Shift Reduction

The previous two steps ensured that the proposed method extracts rich hidden features with less noise while the domain shift between the source and target features still exists. The proposed method calculated the CORAL distance to minimize the domain shift. Before calculating the CORAL distance, one fully connected dense layer with 100 nodes was used to extract the deeper hidden expression $h_{s}$ and $h_{t}$, as shown in (13). They were used to calculate CORAL distance through (4). Significantly, the CORAL distance was designed as one loss function $L_{coral}$ to update the network in the proposed method. Through $L_{coral}$, the encoder parts of the source and target domain are connected and shared to learn domain shift knowledge to detect the faults.

(13) $$\left\{\begin{array}{c} h_{s}=Dense\left(Code_{s}\right)\\ h_{t}=Dense\left(Code_{t}\right) \end{array}\right.$$

### 3.5. Fault Diagnosis

After domain shift reduction, the model learned that domain-invariant features $h_{s}$ would be used to detect the faults. The proposed method for fault diagnosis was trained by minimizing the classification loss $L_{c}$, which we defined as cross-entropy loss, denoted as:

(14) $$L_{c}=-\frac{1}{N}\overset{N-1}{\underset{i=0}{\sum }}\overset{C}{\underset{c=0}{\sum }}y_{i,c}^{s}p_{i,c}^{s}$$

where N denotes the number of samples, C is the faulty types, $y_{i,c}^{s}$ is the $i^{th}$ source sample within label c, and $p_{i,c}^{s}$ is the probability of $y_{i,c}^{s}$. The softmax function maps the final output into one probability vector; the biggest probability is selected as the predicted faulty type.

The final loss for training the model was the combination of CORAL loss $L_{coral}$, reconstruction loss $L_{mse}$, and fault diagnosis classification loss $L_{c}$, as shown in (15), where $L_{coral}$ is to minimize the domain shift, $L_{mse}$ is for the 1D-CNN autoencoder to reduce the noise, and $L_{c}$ is for fault diagnosis. Moreover, α is the domain adaption ratio, and β is the reconstruction ratio. They are two tradeoff parameters to balance FD and others. Particularly, we set α as 0.1 since the main task of the proposed model is FD, which was motivated by [32]; a grid search was designed to find the best parameter β, which was set as 10 and is discussed later. Moreover, the proposed model was trained with the optimizer of Adam.

(15) $$L_{proposed}=\alpha L_{coral}+\beta L_{mse}+L_{c}$$

## 4. Experimental Verification

To verify the effectiveness of the proposed method, we implemented the proposed method based on the operating system of ubuntu 16.04 with intel(R) i7 700 CPU. The programming language was python 3.5, and the deep learning platform was Keras.

### 4.1. Data

Case Western Reserve University (CRWU) bearing data center provides some public faulty bearing data sets, which were utilized to test the proposed method’s performance. We adopted data collected at 12,000 samples per second for drive end bearing and normal experiments. It consisted of four faulty types according to faulty diameter, including 7, 14, 21, and 28 mils under four different loads: 0 power horse (PH), 1 PH, 2 PH, and 3 PH. Each faulty type was caused by three different components of bearing: inner ring (IR), ball, and outer ring (OR) except for 28 mils only caused by IR and ball. Thus, each load of faulty data consisted of 11 = 3 × 3 + 2 faulty types: IR7, Ball7, OR7, IR14, Ball14, OR14, IR21, Ball21, OR21, IR28, Ball28, and one normal type. Therefore, the FD problem was one 12 classification problem.

For simplicity, we integrated the data into four subsets: A, B, C, and D corresponding to 0 PH, 1 PH, 2 PH, and 3 PH for analysis, respectively. Each subset was a long time series processed by the overlap Algorithm 1 to generate training samples, as shown in Figure 2. Primarily, we generated 685 samples for each fault with a length of 2048, which is identical to [5]. That is, each subset consisted of 8220 = 685 × 12 samples. Moreover, one summary subset E that combines A, B, C, and D was built to also analyze. The details of each subset are given in Table 1. Here, rpm is revolutions per minute.

**Figure 3:**
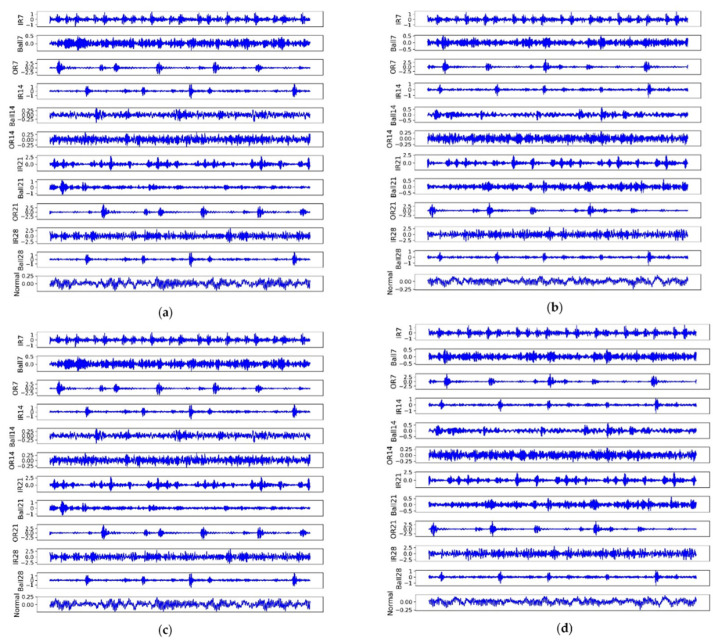
The raw signal for each fault in four subsets (loads). (**a**) Is subset A; (**b**) is subset B; (**c**) is subset C, and (**d**) is subset D.

We give an example for each fault under different loads to illustrate the difficulties for FD, as shown in Figure 3. Although in the same load, each faulty signal shocked fluently and randomly, which increased the difficulty for FD. Primarily, it was difficult to identify (IR7, IR28), (Ball14, OR14), and (OR7, IR14, OR21, OR). Moreover, the same faults displayed differently under different loads, e.g., Ball21 in subset A was far from subset B. In contrast, some faults had high similarities, e.g., Ball28 in subset C was similar to IR14 in subset D.

**Figure 4:**
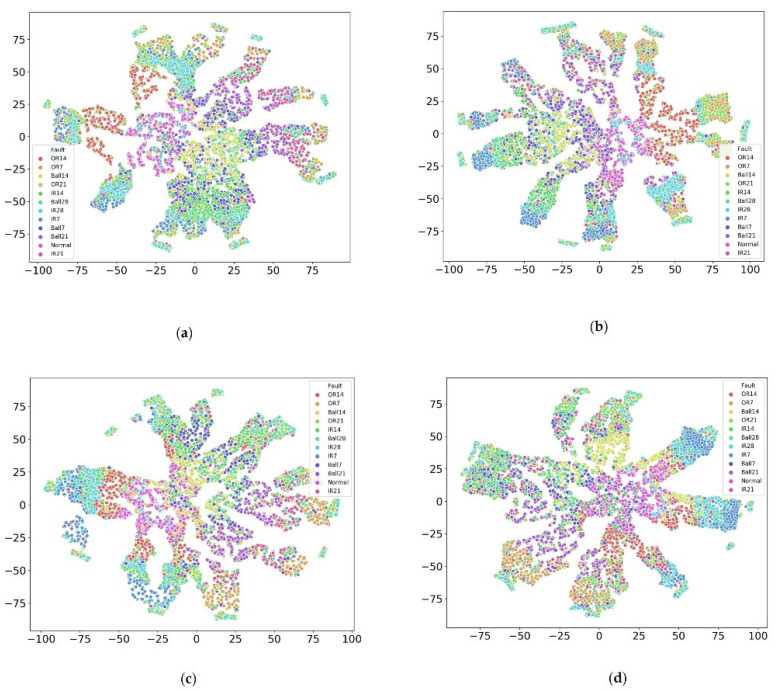
Each fault inner distribution visualization under different loads via t-SNE. (**a**) Is subset A; (**b**) is subset B; (**c**) is subset C, and (**d**) is subset D.

Moreover, we utilized t-distributed stochastic neighbor embedding (t-SNE) technology to see the inner distribution of each fault under different loads, as shown in Figure 4. The results showed that each fault had a different distribution under different loads. For instance, the distribution OR14 for subsets A and B were relatively concentrated while others were not; Ball 7 was distributed concentratedly for subset C and others were distributed sparsely. Moreover, different faults were mixed, inseparable, non-linear, and difficult to identify. Similar to IR28 and Ball 28 in subset A were IR7 and IR28 in subset B, OR14 and normal in Subset C, and IR7 and Ball 28 in subset D. One common finding was that all faults were distributed in a transmitting ring for different loads, which led us to apply deep transfer learning to detect the faults under a complex environment.

Above mentioned characteristics make FD more challenging. To overcome those issues, the proposed model must learn rich distinguishable features and have an excellent transferable capacity as we cannot collect all kinds of faulty data under a complex production environment. Therefore, this manuscript combined a 1D-CNN autoencoder and CORAL to extract rich distinguishable, domain-invariant features with less noise for FD, in which 1D-CNN autoencoder was to extract rich distinguishable features while CORAL was to minimize the domain shift between source and target data.

### 4.2. One Domain Fault Diagnosis

To verify the proposed method’s performance for FD, we first analyzed its performance for one-domain FD, i.e., training and testing the model in the same load. Primarily, we compared the proposed method with both traditional machine learning methods: SVM [6] and RF [8], and current state-of-the-art deep learning-based methods: WDCNN [22], MSFFCNN [25], MDCNN [5], and MSCNN [24]. Particularly, we implemented SVM and RF based on the library of ‘sklearn’ with default settings and strictly reproduced deep learning models according to the given parameters in the papers. For the proposed method, we adopted the 1D-CNN autoencoder for one-domain FD, in which the parameter β was set as 10 to reconstruct the signal, and its influence is discussed later.

**Figure 5:**
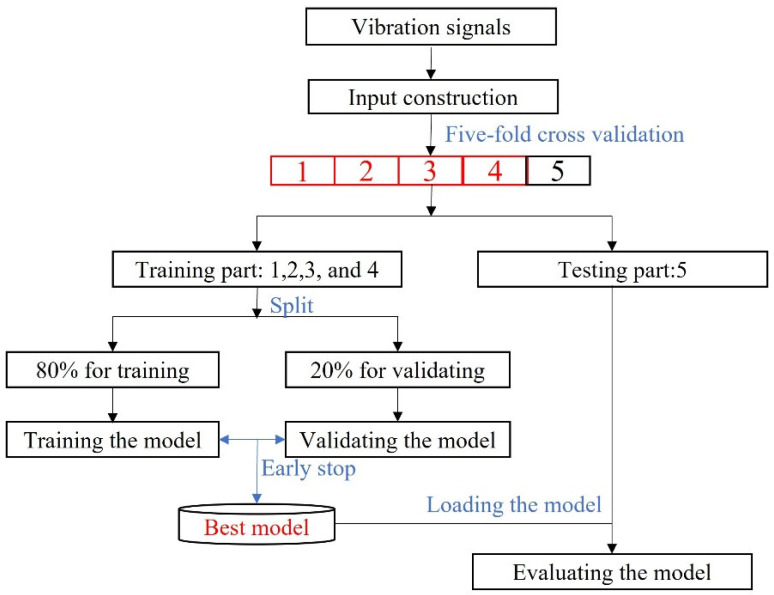
The workflow for one-domain fault diagnosis testing used a five-fold cross-validation approach.

Moreover, each method was fairly verified through a five-fold cross-validation approach, the workflow of which is given in Figure 5. Firstly, the collected vibration signals were processed with Algorithm 1 to generate input matrices (7) and (8). Then, the input and corresponding labels were randomly split into five equal parts. Four of them were used as a training set to train the model, while the rest was for testing. Thirdly, 80% of the training set was used to train the model, while 20% was used as validation data to find the best convergence path with an early stop strategy. Primarily, we set training epochs as 100, and patience was five, i.e., if the validation accuracy was not increased in five continuous epochs, the training process stopped. Then, the model with the highest validation accuracy was saved for evaluating the model on the testing subset. Otherwise, the training process executed until 100 epochs.

**Figure 6:**
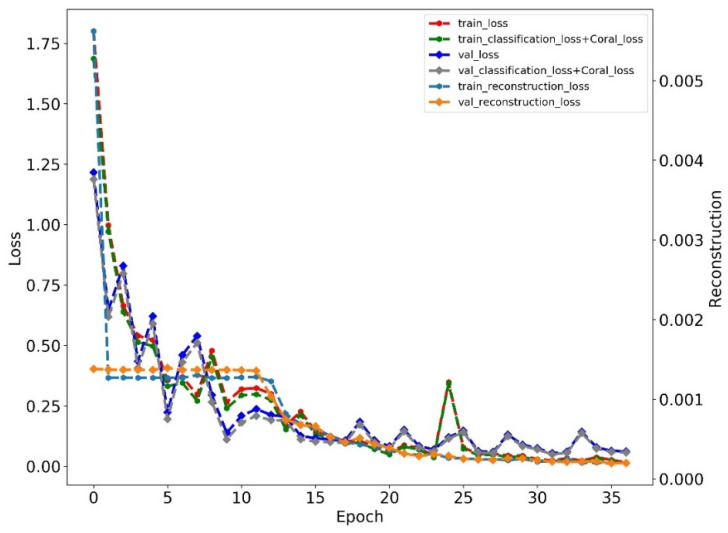
The training loss of the proposed method on subset A.

We give one training loss curve example of the proposed method on subset A, as shown in Figure 6. The results showed that the training process ended at epoch 37, and the training loss mainly was controlled by classification loss and CORAL loss as the reconstruction loss was much less than them.

We adopted the average accuracy of five-fold cross-validation to evaluate each method, as shown in Table 2. The results showed that the proposed method received the highest average accuracy of 99.93% for five subsets with the lowest standard error of 0.07%. It performed the best on subsets B, C, and D. Significantly, the proposed method received an accuracy of 100% on subsets C and D. Moreover, the proposed method did not require any additional inputs. In contrast, MSCNN requires calculating three-scale mean values, and MDCNN requires calculating six statistic indexes and DWT transformed coefficients, and therefore, we saved much time for training the model. Although WDCNN did not require any additional inputs, its accuracy was lower than the proposed method. Another finding was that the traditional machine learning method performed worst without the feature selection operation; their accuracies were lower than 75%. In contrast, deep learning-based methods performed much better, whose accuracies were near 100%. Those methods could be ranked as follows: the proposed method > MSCNN > MDCNN > MSFFCNN > WDCNN > RF > SVM according to the average accuracy of five subsets and standard errors.

### 4.3. Cross-Domain Fault Diagnosis

The previous subsection confirmed the effectiveness of the proposed method for one-domain FD. In practice, collecting all kinds of data is difficult and even not available. Therefore, building one transferable model that trains the model on the source data and performs well on unseen target data is critical and necessary. In this manuscript, we defined twelve transfer learning tasks: A→B, A→C, A→D, B→A, B→C, B→D, C→A, C→B, C→D, D→A, D→B, and D→C to verify the proposed method’s effectiveness, where A→B means training the model on the subset A while testing it on subset B, and so forth.

We compared the proposed method with several machine learning methods and deep learning-based methods, identical to one-domain FD, to illustrate its priority for cross-domain FD. Moreover, we compared it with two domain adaptive methods: WDCNN + AdaBN [23] and the deep adaptive model with MMD (DaMMD) [29]. Notice that we combined the structure of 1D-CNN in the proposed method and MMD for DaMMD to compare fairly.

We trained and tested each comparative method for each task ten times. Each time adopted an early stop strategy to find the best model. The results as the ten-time average accuracy are shown in Table 3. The results indicated that the proposed method outperformed others in the average accuracy of twelve tasks, up to 96.40%. Traditional machine learning methods performed worse for cross-domain FD. Particularly, SVM and RF only obtained 89.58% and 47.47%, respectively, for twelve tasks. The deep learning-based methods could be divided into two groups according to their performance. One includes WDCNN and MDCNN as their accuracy was higher than 90%; another consists of MSFCNN and MSCNN as their accuracy was lower than 90%. For the domain adaptive deep model, WDCNN + AdaBN obtained 94.92% average accuracy for twelve tasks, and DaMMD was 92.68%. Those methods could be ranked as follows: the proposed method > WDCNN + AdaBN > MDCNN > WDCNN > DaMMD > MSFFCNN > MSCNN > SVM > RF. Not supervised, the proposed method showed a significant improvement compared to others. Significantly, it improved by 1.56% compared to WDCNN + AdaBN; 2.01% compared to MDCNN; 4.01% compared to DaMMD; and 103.08% compared to RF.

Moreover, only the proposed method performed better than 90% for all transfer tasks. It received the three highest accuracies for tasks A→D, B→A, and B→D, with accuracies of 95.07%, 99.41%, and 97.10%, respectively. Even though DaMMD obtained the four best accuracies, it was not stable. Primarily, it only received an accuracy of 77.41% for A→D. On the contrary, the proposed method received the lowest standard error of 2.19%, and it was much more robust for cross-domain FD.

**Figure 7:**
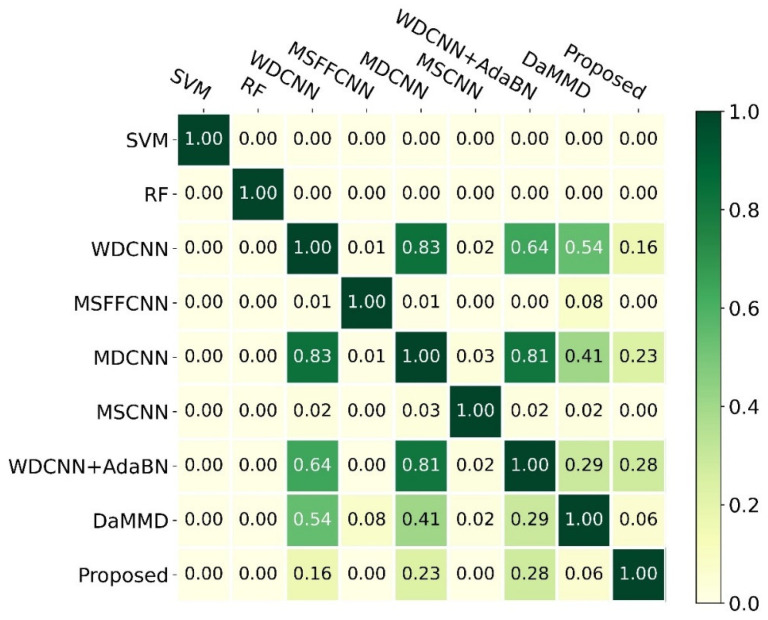
The p-values of the *t*-test for different methods.

To quantify the difference among those methods, we calculated the p-value of the t-test, as shown in Figure 7. Notice that we only kept two decimal places. The findings indicated that the proposed method was significantly different from SVM, RF, MSFFCNN, and MSCNN due to their p-values being much less than 0.05, and it was a little different from DaMMD due to the *p*-value being 0.06. Moreover, there was no significant difference between the proposed method and WDCNN, MDCNN, and WDCNN + AdaBN. However, the proposed method was more accurate and stable. In addition, SVM and RF were significantly different from others.

The above analysis confirmed that the proposed method could detect the fault accurately and robustly under a complex environment using the raw signals without any labeled target domain samples.

### 4.4. The Effectiveness of Each Component

We designed an ablation study to explore the effectiveness of each component in the proposed method. Specifically, we designed a 1D-CNN and 1D-CNN autoencoder to validate the effectiveness of the autoencoder. We designed 1D-CNN+CORAL to validate the effectiveness of CORAL; designed the proposed method to see the effectiveness of the combination of 1D-CNN, autoencoder, and CORAL; and designed the proposed method with two decoders (Proposed_two_) to verify their effectiveness. The results of the ablation study are shown in Table 4. The results showed that 1D-CNN obtained 92.77% average accuracy for twelve tasks, higher than DaMMD. The application of the autoencoder improved the accuracy from 92.77% to 92.79. Moreover, the usage of CORAL improved the accuracy by 0.05%, which could be calculated by comparing the 1D-CNN and 1D-CNN+CORAL. By combining 1D-CNN, autoencoder, and CORAL, the average accuracy was 96.40%, which improved by 3.63% compared to 1D-CNN. Although the Proposed_two_ won serval cases such as A→C, A→D, and B→D**,** its average accuracy for twelve tasks was lower than the proposed method while its standard error was higher. Particularly, the utilization of the decoder part in the target data decreased the average accuracy by 0.19%. In addition, it increased the model’s size, which was the reason we adopted the decoder part in the source data for the proposed method.

We calculated the contribution ratio $C_{ration}=\frac{Ip_{c}}{Ip_{total}}$ for each component, where $Ip_{c}$ denotes the improvement of each component and $Ip_{total}$ is the actual improvement of the proposed method. The results showed that autoencoder contributed to a 0.56% improvement while CORAL contributed 1.38%. The combination of the autoencoder and CORAL contributed 98.07%.

### 4.5. Anti-Noise Testing

**Figure 8:**
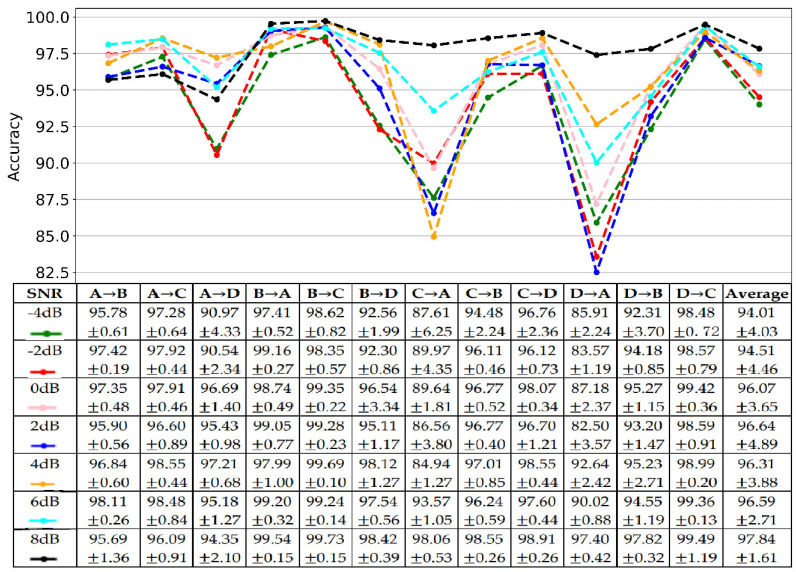
The results of antinoise testing.

The data collected from the natural production environment convolves some noises, which increases the difficulty for FD. It requires the proposed model to have an excellent anti-noise capacity. We tested the proposed method’s anti-noise capacity on twelve transfer tasks with simulated white noise. Particularly, different intensity white noises were added into raw signals to train and test the model. The intensity of white noise was measured with the signal-noise ratio (SNR), defined as (16), where $p_{s\;}$and $p_{w}$ are the power of the signal and white noise, respectively, whose unit is the decibel (dB). The testing results with different SNR ranged from −4 dB to 8 dB, as shown in Figure 8. The results showed that the proposed method has an excellent anti-noise capacity. Significantly, the proposed method’s average accuracy for twelve transfer tasks was higher than 94%. The lowest accuracy was 94.01% under the noise of −4 dB, while the highest was 97.84% under an 8 dB noisy environment.

(16) $$\mathrm{SNR}=10log\frac{p_{s\;}}{p_{w}}$$

Moreover, the model’s performance increased with the SNR, which means that the model performs better for FD with less noise. Moreover, all cases showed the same trend. Mainly, the proposed method performed worse for A→D, C→A, and D→A, while it performed better for B→A, B→C, C→D, and D→C. Another finding was that the proposed method with a bit of noise, especially at 6 dB (96.59%) and 8 dB (97.84%), performed better than without noise (96.40%). It indicated that adding a little noise could help the model learn more distinguishable features.

**Figure 9:**
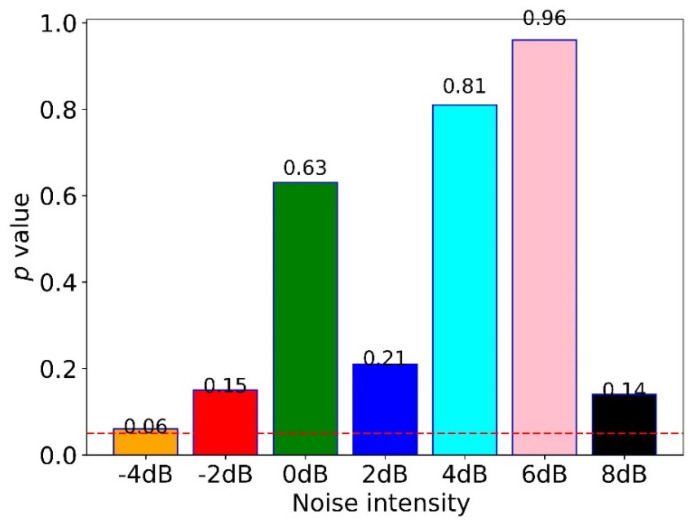
The *p*-value of the *t*-test for the proposed method under noisy and non-noisy environments.

To quantify the difference between the proposed method under noisy and non-noisy environments, we calculated the p-value of the t-test, as shown in Figure 9. The results indicated no significant difference between non-noisy and noisy environments as their p-values were higher than 0.05 (the red dotted line). Moreover, the proposed method with noise performed better than all comparative methods, except for WDCNN + AdaBN which performed a little better than the proposed method under the noise of −4 dB and −2 dB; WDCNN and MDCNN performed a little better than the proposed method at −4 dB.

### 4.6. The Effectiveness of Tradeoff Parameter β

**Figure 10:**
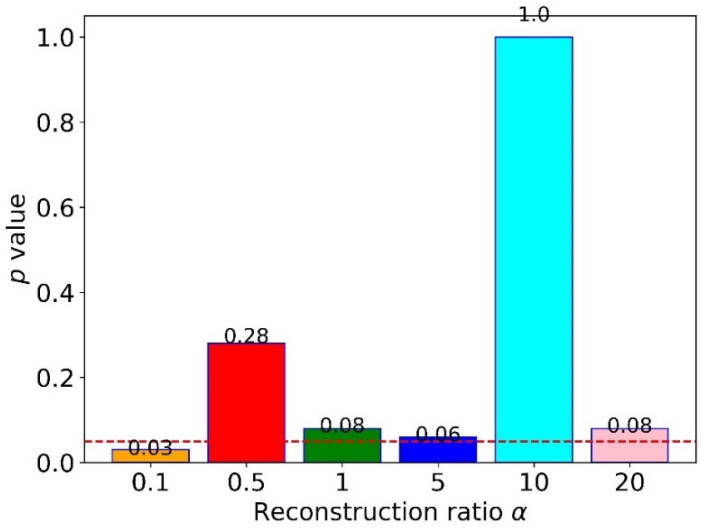
The p-values of the *t*-test between a reconstruction ratio of ten and others.

To explore the influence of the reconstruction ratio β, we designed five sub-experiments with different α for cross-domain FD, where α was from 0.1 to 20. The results for twelve tasks are shown in Table 5. The results showed no apparent patterns in different β, but the proposed method obtained the highest accuracy when we set it as 10. Moreover, all five sub-experiments’ accuracies were higher than 90%, better than MSCNN and MSFFCNN. To quantify the difference between 10 and others, we calculated the p-value of the t-test, as shown in Figure 10. The findings indicated that only the reconstruction ratio of 0.1 had a significant difference from 10. In contrast, others did not, which could be calculated by comparing their p-values with 0.05 (dotted line in Figure 10). This finding suggested that setting a slightly big reconstruction ratio is better, especially setting ten as the reconstruction ratio.

## 5. Discussion

FD plays a critical role in building a smart factory, which can help the manager find the fault timely to avoid accidents and improve the system’s efficiency. Learning-based methods that can extract the feature automatically from raw signals have been widely applied for FD. However, most of them assume that we can collect sufficient historical data to train the model, which is not easy in practice. Moreover, they assume that the source and target data have the same distribution, decreasing the FD’s performance.

This manuscript proposed a novel, domain adaptive, and effective deep model based on 1D-CNN for cross-domain FD to solve the above issues, as shown in Figure 1. The 1D-CNN autoencoder was developed to extract rich, less-noisy hidden features from raw signals. The CORAL processed the extracted features from the source and target data to minimize domain shift. Therefore, the proposed method could accurately detect the faults on unseen target domain data using a model trained on source domain data.

To validate the effectiveness of the proposed method, we compared the proposed method with some learning-based methods on CRWU bearing data sets. Significantly, we verified the one-domain FD’s capacity using a five-fold cross-validation approach, as shown in Figure 5. The comparative analysis confirmed its effectiveness for one-domain FD, as shown in Table 2. Significantly, the proposed method won three times for five subsets and received the highest average accuracy.

To validate the effectiveness of the proposed method for cross-domain FD, we compared the proposed method with other leading methods on twelve transfer tasks. The experimental results showed that the proposed method outperformed others, as shown in Table 3. Significantly, the proposed method obtained 96.40% average accuracy. Moreover, only the proposed method’s accuracy was higher than 90% for all tasks; it obtained the three highest accuracies for tasks A→D, B→A, and B→D, with accuracies of 95.07%, 99.41%, and 97.10%, respectively. From the view of standard error, we could conclude that the proposed method has good robustness, with a standard error of 2.19%. The t-test results indicated that the proposed method was significantly different from SVM, RF, MSFFCNN, and MSCNN due to their p-values being much less than 0.05, and it was slightly different from DaMMD due to the *p*-value being 0.06, as shown in Figure 7. Moreover, there was no significant difference between the proposed method and WDCNN, MDCNN, and WDCNN + AdaBN.

An ablation study was designed to validate each component’s effectiveness in the proposed method. The results showed that 1D-CNN obtained 92.77% average accuracy for twelve tasks, which was higher than DaMMD; the application of the autoencoder improved the accuracy by 0.02%, and the CORAL improved the accuracy by 0.05%. By combining 1D-CNN, autoencoder, and CORAL, the average accuracy increased up to 96.40%, as shown in Table 4.

The anti-noise testing results showed that the proposed method is not sensitive to noise, as shown in Figure 8. Significantly, the average accuracy of the proposed method was higher than 94%. It obtained the lowest accuracy of 94.01%, and the highest was 97.84%. Additionally, the model’s performance increased with the SNR, which means that the model performs better for FD with less noise. Moreover, the results showed that adding a little noise could increase the performance of the proposed method. In addition, there was no difference between non-noisy and noisy environments (−4 dB to 8 dB) for the proposed method, which is shown in Figure 9.

To explore the effect of the reconstruction ratio β in the proposed method, we designed six sub-experiments with different β from 0.1 to 20, as shown in Table 5. The results showed that the proposed method performed the best when setting β as 10. Moreover, the proposed method was not sensitive to the reconstruction ratio when setting it a little big, which is shown in Figure 10.

The proposed method has 357,161 parameters and takes up to 4.4 megabytes (MB), and each training step takes three milliseconds (ms). It is easy to deploy to all kinds of clients for real-time FD, and it takes around 0.7 ms to detect one sample.

As discussed above, different reconstruction ratios influence the FD’s performance. This manuscript selected the best parameter manually, which was time-consuming. In the future, we will develop an automatic structure to search for a better reconstruction ratio from a wide range. Moreover, the authors will test the proposed method’s generalizability on other time series data sets.

## 6. Conclusions

This manuscript proposed a novel, lightweight, and domain adaptive framework based on 1D-CNN for accurately detecting the faults under complex environments. Particularly, 1D-CNN with an autoencoder structure was designed to extract the rich hidden features with less noise from raw signals; CORAL processed the extracted features to minimize the domain shift. Therefore, the proposed could extract rich, domain-invariant features with less noise for FD. The massive comparative experiments indicated that the proposed method could accurately detect the faults under a complex environment only using raw signals without labeled target samples. Significantly, the proposed method obtained near 100% accuracy for one-domain FD and 96% for cross-domain FD. Moreover, the proposed method has a good anti-noise capacity, and the noise almost does not influence the proposed method. On the contrary, adding little noise can improve its performance.

In the future, we will design an automatic structure based on deep reinforcement learning (DRL) to search for the best reconstruction ratio under the proposed framework for FD more accurately. Additionally, we will verify its generalizability on other time series data sets.

## Figures and Tables

**Table 1:** Data description.

Subset	Samples	Faulty Types	Conditions (Load, Speed)
A	8220	IR7, Ball7, OR7, IR14, Ball14, OR14, IR21, Ball21, OR21, IR28, Ball28, and normal	0 PH, 1797 rpm
B	8220	IR7, Ball7, OR7, IR14, Ball14, OR14, IR21, Ball21, OR21, IR28, Ball28, and normal	1 PH, 1772 rpm
C	8220	IR7, Ball7, OR7, IR14, Ball14, OR14, IR21, Ball21, OR21, IR28, Ball28, and normal	2 PH, 1750 rpm
D	8220	IR7, Ball7, OR7, IR14, Ball14, OR14, IR21, Ball21, OR21, IR28, Ball28, and normal	3 PH, 1730 rpm
E	32,880	IR7, Ball7, OR7, IR14, Ball14, OR14, IR21, Ball21, OR21, IR28, Ball28, and normal	0,1,2,3 PH 1797, 1772, 1750, 1730 rpm

**Table 2:** The comparative results for one-domain FD (%).

Method	A	B	C	D	E	Average
SVM [6]	66.40 ± 0.39	71.09 ± 0.97	67.81 ± 1.05	70.82 ± 0.92	65.93 ± 0.27	68.41 ± 2.17
RF [8]	71.56 ± 3.72	75.21 ± 1.28	74.31 ± 0.91	76.50 ± 0.39	74.26 ± 0.48	74.37 ± 1.62
WDCNN [22]	99.34 ± 0.40	99.04 ± 0.14	99.88 ± 0.10	99.91 ± 0.08	99.70 ± 0.15	99.57 ± 0.34
MSFFCNN [25]	99.66 ± 0.30	99.57 ± 0.22	**100 ± 0.0**	98.99 ± 0.02	99.80 ± 0.29	99.60 ± 0.34
MDCNN [5]	99.81 ± 0.30	**99.94 ± 0.0**	99.96 ± 0.05	**100.0 ± 0.0**	99.90 ± 0.05	99.92 ± 0.06
MSCNN [24]	**99.94 ± 0.01**	99.76 ± 0.06	**100 ± 0.0**	**100.0 ± 0.0**	**99.96 ± 0.12**	**99.93 ± 0.09**
1D-CNN autoencoder	99.82 ± 0.19	**99.94 ± 0.11**	**100 ± 0.0**	**100.0 ± 0.0**	99.90 ± 0.05	**99.93 ± 0.07**

**Table 3:** The comparative results for cross-domain FD (%).

Method	A→B	A→C	A→D	B→A	B→C	B→D	C→A	C→B	C→D	D→A	D→B	D→C	Average
SVM [6]	55.80	53.89	54.45	56.14	59.83	67.06	52.12	66.81	68.64	52.23	66.62	61.35	59.58 ± 6.04
RF [8]	48.81 ± 0.52	46.70 ± 0.67	45.82 ± 0.52	48.13 ± 0.41	50.34 ± 0.61	48.59 ± 0.32	44.96 ± 0.56	49.35 ± 0.34	50.05 ± 0.61	42.34 ± 0.54	45.96 ± 0.71	48.55 ± 0.73	47.47 ± 2.26
WDCNN [22]	**97.35** **± 3.03**	96.12 ± 4.32	92.86 ± 7.01	97.16 ± 1.21	99.25 ± 2.03	97.38 ± 2.13	92.45 ± 3.15	97.46 ± 1.35	97.11 ± 3.12	82.89 ± 7.45	85.52 ± 6.32	93.09 ± 7.14	94.05 ± 4.88
MSFFCNN [25]	91.98 ± 9.12	93.25 ± 7.13	85.07 ± 8.32	92.94 ± 5.00	95.29 ± 5.01	90.24 ± 6.12	81.02 ± 12.11	86.80 ± 12.35	85.12 ± 14.02	85.35 ± 5.01	86.50 ± 7.12	92.08 ± 8.31	88.80 ± 4.70
MDCNN [5]	96.76 ± 2.01	94.91 ± 2.13	94.32 ± 4.12	96.22 ± 2.03	**99.84** **± 1.01**	98.90 ± 1.03	93.79 ± 1.23	97.02 ± 2.23	98.22 ± 3.00	86.05 ± 6.08	84.28 ± 8.75	93.56 ± 6.44	94.50 ± 4.59
MSCNN [24]	86.85 ± 5.18	81.85 ± 4.67	80.75 ± 9.26	85.52 ± 5.24	97.87 ± 2.29	92.12 ± 3.24	81.13 ± 5.78	93.60 ± 0.59	**99.36** **± 0.59**	72.65 ± 5.94	77.92 ± 4.00	86.80 ± 5.31	86.18 ± 7.83
WDCNN + AdaBN [23]	91.97 ± 2.81	**98.02** **± 0.98**	89.37 ± 4.43	94.98 ± 2.85	99.66 ± 1.03	97.93 ± 0.90	94.30 ± 1.38	97.71 ± 0.36	98.14 ± 1.19	87.01 ± 3.32	91.97 ± 2.81	98.02 ± 0.98	94.92 ± 3.87
DaMMD [29]	90.03 ± 1.42	87.66 ± 1.43	77.41 ± 2.05	97.17 ± 0.18	95.09 ± 0.74	90.02 ± 2.35	**95.73** **± 0.48**	**97.78** **± 0.16**	92.35 ± 0.80	**93.28** **± 0.33**	96.91 ± 0.22	**98.69** **± 0.33**	92.68 ± 5.69
Proposed	96.88 ± 0.53	96.06 ± 0.69	**95.07** **± 1.21**	**99.41** **± 0.22**	99.47 ± 0.27	**97.10** **± 0.80**	93.00 ± 1.20	96.40 ± 0.43	97.51 ± 0.24	91.95 ± 0.75	95.68 ± 0.79	98.25 ± 0.59	**96.40** **± 2.19**

**Table 4:** The results of the ablation study.

Method	A→B	A→C	A→D	B→A	B→C	B→D	C→A	C→B	C→D	D→A	D→B	D→C	Average
1D-CNN	94.49 ± 4.29	95.27 ± 3.27	87.53 ± 6.02	95.29 ± 4.14	99.94 ± 0.08	92.61 ± 3.08	92.08 ± 2.25	96.56 ± 1.72	93.82 ± 6.52	86.14 ± 4.22	86.25 ± 4.48	93.27 ± 6.44	92.77 ± 4.05
1D-CNN autoencoder	94.91 ± 0.04	95.47 ± 0.04	88.80 ± 0.05	95.53 ± 0.04	**99.81** **± 0.00**	93.64 ± 0.05	93.15 ± 0.04	97.40 ± 0.01	94.42 ± 0.04	87.64 ± 0.06	87.01 ± 0.04	93.70 ± 0.07	92.79 ± 4.98
1D-CNN + CORAL	92.52 ± 1.77	83.81 ± 2.48	81.15 ± 3.31	96.74 ± 0.74	94.64 ± 1.16	87.76 ± 1.41	**93.31** **± 0.52**	**98.29** **± 0.15**	94.95 ± 0.57	**93.34** **± 0.61**	**96.96** **± 0.12**	**98.60** **± 0.21**	92.83 ± 5.43
Proposed_two_	96.53 ± 0.46	**96.64** **± 1.17**	**97.23** **± 0.40**	**99.60** **± 0.22**	**99.64** **± 0.07**	**98.78** **± 0.34**	91.28 ± 2.50	93.71 ± 2.41	97.50 ± 0.37	89.21 ± 0.74	96.48 ± 0.48	97.99 ± 1.11	96.21 ± 3.11
Proposed	**96.88** **± 0.53**	96.06 ± 0.69	95.07 ± 1.21	99.41 ± 0.22	99.47 ± 0.27	97.10 ± 0.80	93.00 ± 1.20	96.40 ± 0.43	**97.51** **± 0.24**	91.95 ± 0.75	95.68 ± 0.79	98.25 ± 0.59	**96.40** **± 2.19**

**Table 5:** The effectiveness of reconstruction ratio β.

β	A→B	A→ **C**	A→ **D**	B→ **A**	B→C	B→D	C→A	C→B	C→D	D→A	D→B	D→C	Average
0.1	94.42 ± 0.57	93.82 ± 0.18	85.98 ± 2.07	96.85 ± 0.67	96.49 ± 0.87	88.76 ± 1.99	87.35 ± 2.37	96.20 ± 0.80	96.74 ± 0.18	77.58 ± 1.58	91.34 ± 1.54	98.05 ± 0.52	91.97 ± 5.82
0.5	94.48 ± 0.50	**96.26** **± 0.29**	94.19 ± 1.46	98.12 ± 0.34	98.00 ± 0.44	94.86 ± 0.70	91.96 ± 1.11	95.76 ± 0.63	97.13 ± 0.52	89.56 ± 1.87	94.27 ± 0.72	**98.73** **± 0.21**	95.28 ± 2.56
1	93.91 ± 3.27	95.55 ± 3.87	94.96 ± 1.57	95.63 ± 0.88	97.04 ± 0.51	93.69 ± 1.60	90.07 ± 4.06	95.03 ± 0.24	96.71 ± 0.60	84.72 ± 1.62	94.05 ± 0.41	98.25 ± 0.31	94.13 ± 3.45
5	94.01 ± 0.91	94.70 ± 0.50	95.64 ± 2.14	98.03 ± 0.53	96.72 ± 0.43	93.85 ± 1.84	91.36 ± 5.70	92.57 ± 0.80	91.09 ± 2.45	95.09 ± 4.64	94.13 ± 2.05	97.47 ± 0.17	93.86 ± 2.17
10	**96.88** **± 0.53**	96.06 ± 0.69	**95.07** **± 1.21**	**99.41** **± 0.22**	**99.47** **± 0.27**	**97.10** **± 0.80**	**93.00** **± 1.20**	**96.40** **± 0.43**	**97.51** **± 0.24**	**91.95** **± 0.75**	**95.68** **± 0.79**	98.25 ± 0.59	**96.39** **± 2.19**
20	95.31 ± 0.79	95.93 ± 0.65	87.59 ± 3.11	99.18 ± 4.58	97.76 ± 1.08	88.80 ± 1.99	92.93 ± 1.25	96.37 ± 0.31	94.02 ± 1.71	83.10 ± 1.97	93.98 ± 0.88	98.01 ± 0.53	93.58 ± 4.60

## Data Availability

The data are from CWRU bearing data center.
